# Crystal structure of the second extracellular domain of human tetraspanin CD9: twinning and diffuse scattering

**DOI:** 10.1107/S2414314622008525

**Published:** 2022-09-23

**Authors:** Viviana Neviani, Martin Lutz, Wout Oosterheert, Piet Gros, Loes Kroon-Batenburg

**Affiliations:** a Department of Chemistry, Structural Biochemistry, Bijvoet Centre for Biomolecular Research, Faculty of Science, Utrecht University, Utrecht, The Netherlands; University of Manchester, United Kingdom

**Keywords:** twinning, diffuse scattering, tetraspanin CD9_EC2_, raw data

## Abstract

Remarkable features are reported in the diffraction pattern produced by a crystal of the second extracellular domain of tetraspanin CD9 (deemed CD9_EC2_), the structure of which has been described previously [Oosterheert *et al.* (2020 ▸), *Life Sci. Alliance*, **3**, e202000883]. CD9_EC2_ crystallized in space group *P*1 and was twinned. Two types of diffuse streaks are observed. The stronger diffuse streaks are related to the twinning and occur in the direction perpendicular to the twinning interface. It is concluded that the twin domains scatter coherently as both Bragg reflections and diffuse streaks are seen. The weaker streaks along c* are unrelated to the twinning but are caused by intermittent layers of non-crystallographic symmetry related molecules. It is envisaged that the raw diffraction images could be very useful for methods developers trying to remove the diffuse scattering to extract accurate Bragg intensities or using it to model the effect of packing disorder on the molecular structure.

## Abstract 2

Remarkable features are reported in the diffraction pattern produced by a crystal of tetraspanin CD9_EC2_.

## Graphical abstract

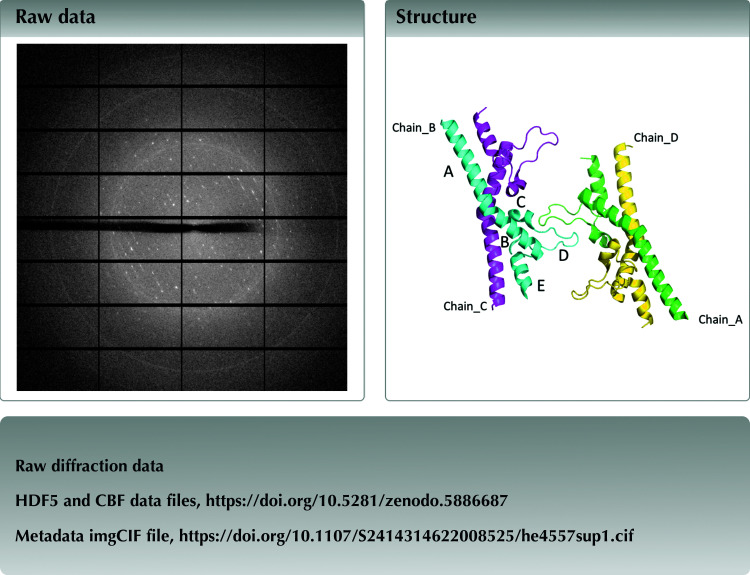

## Data processing and refinement

**Figure 1:**
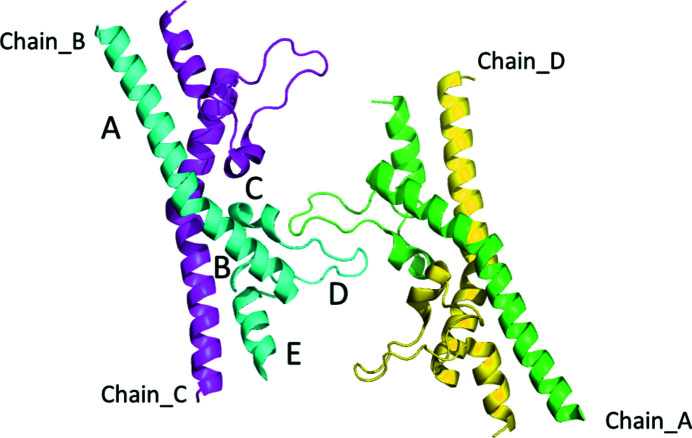
Asymmetric unit of the twinned CD9_EC2_ crystal, coloured by protein chain. Domains are labelled *A*–*E* in the cyan-coloured chain. The *D* loop is flexible as follows from comparison of different structures involving CD9_EC2_ and structures of EC2 domains from other tetraspanins (Oosterheert *et al.*, 2020 ▸). The direction of view is approximately along the non-crystallographic (NCS) twofold axis that coincides with the twofold twin axis a* + b* (see text). The NCS operation transforms chain *A* into chain *B*, and chain *C* into chain *D*.

This letter gives a detailed description of the raw diffraction data that were used for analysis and structure determination of the second extracellular domain of tetraspanin CD9 (CD9_EC2_) as previously reported (Oosterheert *et al.*, 2020 ▸). The raw diffraction images show streaked diffuse scattering, and this feature is detailed here to serve as an example for archiving and re-analysis of raw diffraction data. CD9_EC2_ crystallized in space group *P*1, has four molecules in the asymmetric unit, arranged as a dimer of domain-swapped dimers (Fig. 1 ▸). The crystal was non-merohedrally twinned with a twofold rotation about a* + b* as the twinning operation.

With the *EVAL* software suite (Schreurs *et al.*, 2010 ▸) two lattices could be indexed and reflections of the largest domain were integrated while overlapping reflections of the second domain could be largely deconvoluted, leaving only 21.5% of reflections overlapping.

Initial de-twinning was performed with the *TWINABS* software (Sheldrick, 2009 ▸) based on observed structure factors. These data were used for structure solution by molecular replacement as described previously (Oosterheert *et al.*, 2020 ▸). Final detwinning was based on calculated structure factors and final refinement rounds in *Refmac*5 yielded *R*_work_/*R*_free_ = 23.9/27.9%.

Structural details can be found in (Oosterheert *et al.*, 2020 ▸) and in the Protein Data Bank under the accession code 6rlr. Data collection details and statistics are listed in Table 1 ▸.

## Data description

**Figure 2:**
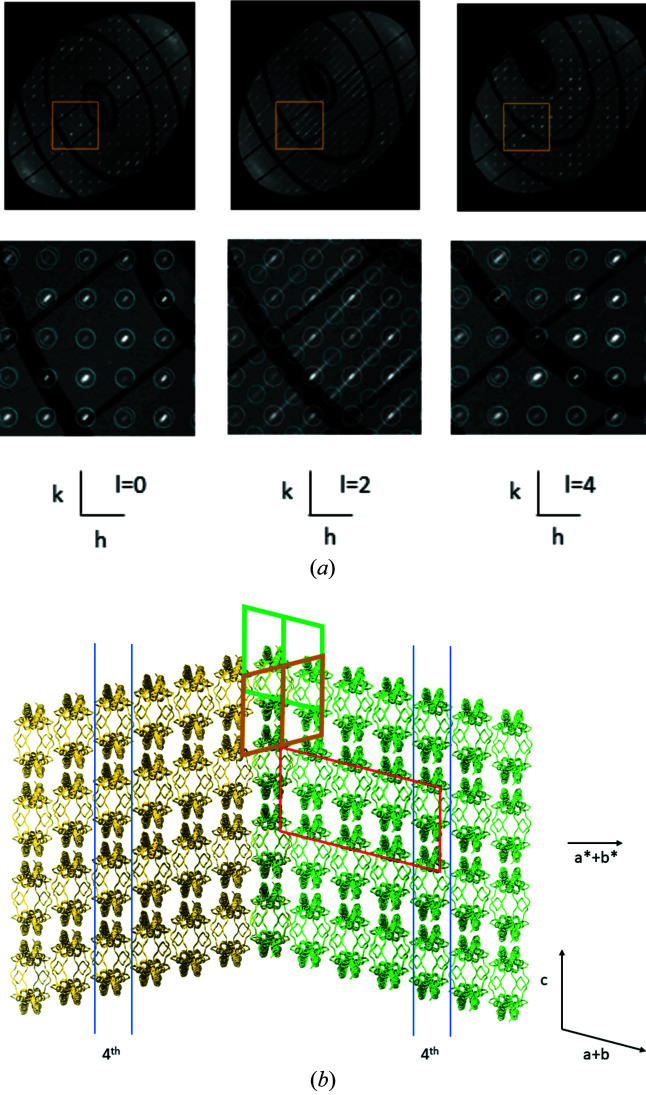
Twinned CD9_EC2_ crystal. (*a*) Reciprocal space reconstructions, *hk*0, *hk*2 and *hk*4. The lower panels are zoomed in at the area of the yellow box, where Bragg reflections originating from lattice 1 and 2 are coloured white and cyan, respectively. In slice *hk*2, streaks along a* + b* are evident. All slices *hk*(*l* = 4*n*) are ordered and the spots indexed by the two matrices nearly overlap, while in slices *hk*(*l* = 4*n* + 2) they have maximum separation. (*b*) Green and gold structures represent the two twin domains in the crystal. The second domain is rotated by 180° around a* + b* with respect to the first. The twin interface is the plane in the middle with base vectors **c** and **a − b** for either lattice. In the figure, molecules of the two domains are overlayed on this layer. The 180° twin rotation applied to domain 1 causes chains *A* and *C* of the molecules in domain 1 (green) to superimpose on chains *B* and *D* of domain 2 (gold), respectively. Starting from the interface the 4th layers (between the blue lines) in the two domains are the same and form an ordered array. A super cell (red) can be constructed with transformation matrix (1,−1,0/0,0,1/−2,−2,1) on which the two twin lattices overlap. The consequence is that in reciprocal space reflections of the twin lattices overlap for every *l* = 4*n*.

Data were collected at Diamond Light Source (DLS) beamline I-04, in total 3600 images in 0.1° fine sliced mode using a single rotation axis. The diffraction data were written in HDF5 format. Since, at the time of data processing, *EVAL* could not read the HDF5 files, they were converted by a tool at DLS to CBF format, using mini-cbf headers, and these were processed by *EVAL*. Both HDF5 (.h5) and CBF (.cbf) data files have been deposited in Zenodo. Indexing of peaks in the diffraction data with *DIRAX* (Duisenberg, 1992 ▸) indicated non-merohedral twinning of the crystal with a twofold rotation around the a* + b* diagonal as the twinning operation (Fig. 2 ▸). Concurrent with twinning, diffuse streaks are seen in the diffraction. Diffraction images were mapped to reciprocal space, to a resolution of 4 Å, using *IMG2HKL* in *EVAL* (Schreurs *et al.*, 2010 ▸), first by merging images in groups of 5, and then carefully redistributing intensities while correcting for Lorentz and polarization factors. The reciprocal space map was merged using Laue symmetry −1. The reciprocal space map is available as a *CCP*4 map (https://doi.org/10.5281/zenodo.6961763), that can *e.g.* be viewed with *Chimera* (Pettersen *et al.*, 2004 ▸). Fig. 2 ▸(*a*) shows three sections through reciprocal space on an *hkl* grid, where evident streaks are running in the a* + b* direction, particularly in the *hk*2 section.

In the following we draw conclusions on the origins of the diffuse scattering features we observed. A variety of diffuse scattering features in macromolecular crystals of different origin is discussed by Glover *et al.* (1991 ▸). For an extensive treatment of diffuse scattering in proteins, we refer to the paper on the Gag protein by Welberry *et al.* (2011 ▸). Geo­metrical frustration of the packing of two molecular configurations of the Gag protein led to circular diffuse scattering features. The origin of our diffuse scattering of CD9_EC2_, though, is different; we only have streaked diffuse scattering that is caused by stacking disorder of layers. We summarize here what we concluded in the Oosterheert paper on the origin of the diffuse scattering (see Fig. 2 ▸ for details).

The twinning interface is a layer with base vector **c** and **a − b**. The two twin domains each grow from this interface along their respective a* + b* directions. For every fourth layer the two structures exactly overlap.

Reflections can be indexed on a so-called stacking lattice (Dornberger-Schiff, 1956 ▸; Lutz & Kroon-Batenburg, 2018 ▸) with dimension 1/4*c*. On this lattice the twin structure is completely ordered and as a result the reciprocal space slices at l = 4*n* have only ordered Bragg spots.

All the other slices have streaks in a* + b* which is the direction of the packing disorder.

A twofold NCS (non-crystallographic symmetry) operation transforms the independent molecules into one of the others; the corresponding axis co-aligns with twin axis a* + b*. Chain *A* superposes with chain *B* (r.m.s.d. 0.482 Å) and chain *C* with chain *D* (r.m.s.d. 0.460 Å). The CD9_EC2_ molecule has a flexible *D* loop (see Fig. 1 ▸, where the *D* loop is marked for chain *B*). In the structure this loop is located at the twin interface. A crystal consisting of small twin domains is coherently scattering over a length scale determined by the coherence length of the X-rays (see Thompson, 2017 ▸ for a discussion on order–disorder and twinning). This is the case for our crystal, which gives rise to both Bragg peaks and diffuse streaks [Fig. 2 ▸(*a*)].

**Figure 3:**
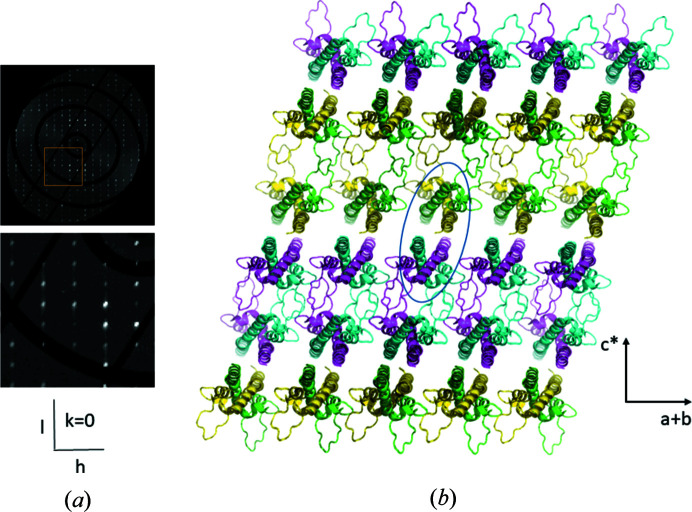
Diffuse scattering along c*. (*a*) Reciprocal space reconstruction of *h*0*l* layer and zoomed in at the area of the yellow box. Intensities are on the same scale as in Fig. 2 ▸(*a*). Streaks are observed along c*, but much weaker than those along a* + b*. (*b*) Model of a single domain in which one layer is replaced by a rotation copy generated by the NCS rotation operation, which coincides with the twinning, *i.e.* 180° rotation around a* + b*. The colouring of the chains is the same as in Fig. 1 ▸, but the asymmetric unit used here (indicated by an ellipse) is such that the loops are on the outside. Inclusion of a layer of rotated copies will disrupt the periodicity perpendicular to the *ab* layer which is the c* direction, leading to diffuse streaks in the diffraction pattern.

It was noticed by a reviewer that streaks are also seen in the c* direction. This is indeed the case, and they occur for every *hkl* layer containing c*, but are significantly weaker than the a* + b* streaks [Fig. 3 ▸(*a*)]. The origin of the diffuse streaks lies within a single domain and is unrelated to the twinning. Our reasoning is that due to NCS, local rotation of molecules can occur, without serious clashes. It may be that within a single domain, a rotated copy of an entire *ab* layer is included in the lattice [see Fig. 3 ▸(*b*)], which is conceivable because the molecules are packed through the *D* loops. This leads to disruption of periodicity in stacking of *ab* layers and to diffuse streaks in the c* direction.

We present here the raw diffraction data and the most likely explanation for the diffuse features. Any interested researcher can generate detailed models of disorder, calculate the diffuse scattering and compare them with our data.

## Supplementary Material

Metadata imgCIF file. DOI: 10.1107/S2414314622008525/he4557img.cif

CheckCIF for raw data report. DOI: 10.1107/S2414314622008525/he4557img_check.pdf

HDF5 and CBF data files: https://doi.org/10.5281/zenodo.5886687

## Figures and Tables

**Table 1:** Experimental details Data for the highest resolution shell are given in parentheses.

Raw data	
DOI	https://doi.org/10.5281/zenodo.5886687
Data archive	Zenodo
Data format	HDF5 and CBF
	
Data collection	
Beamline	Diamond I04
Detector type	EIGER 16M
Radiation type	Synchrotron X-ray source
Wavelength (Å)	0.979491
Beam centre (mm)	−166.87, 172.50
Detector axis	−*Z*
Detector distance (mm)	287.22
Pixel size (mm)	0.075 × 0.075
No. of pixels	4148 × 4362
No. of scans	1
Scan axis	ω, *X*
Start angle, increment per frame (°)	0.0, 0.1
Scan range (°)	360
No. of frames	3600
Exposure time per frame (s)	0.01
	
Crystal and refinement data	
Resolution range (Å)	29.02–2.0 (2.07–2.0)
Space group	*P*1
Cell dimensions *a*, *b*, *c* (Å)	39.986, 39.998, 63.643
Cell angles α, β, γ (°)	80.39, 76.29, 68.15
Total no. of reflections	76175 (6985)
No. of unique reflections	22863 (2180)
Completeness (%)	95.6 (93.0)
Multiplicity	3.4 (3.4)
*I*/σ(*I*)	4.8 (0.8)
*R*_merge_	0.10 (1.15)
*R*_p.i.m._	0.07 (0.73)
CC_1/2_	0.998 (0.322)

## References

[bb5] Dornberger-Schiff, K. (1956). *Acta Cryst.* **9**, 593–601.

[bb6] Duisenberg, A. J. M. (1992). *J. Appl. Cryst.* **25**, 92–96.

[bb900] Glover, I. D., Harris, G. W., Helliwell, J. R. & Moss, D. S. (1991). *Acta Cryst.* B**47**, 960–968.

[bb7] Lutz, M. & Kroon-Batenburg, L. M. J. (2018). *Croat. Chem. Acta*, **91**, 289–298.

[bb9] Oosterheert, W., Xenaki, K. T., Neviani, V., Pos, W., Doulkeridou, S., Manshande, J., Pearce, N. M., Kroon-Batenburg, L. M. J., Lutz, M., van Bergen en Henegouwen, P. M. P. & Gros, P. (2020). *Life Sci. Alliance*, **3**, e202000883. 10.26508/lsa.202000883 PMC7536822 32958604

[bb10] Pettersen, E. F., Goddard, T. D., Huang, C. C., Couch, G. S., Greenblatt, D. M., Meng, E. C. & Ferrin, T. E. (2004). *J. Comput. Chem.* **25**, 1605–1612. 10.1002/jcc.20084 15264254

[bb11] Schreurs, A. M. M., Xian, X. & Kroon-Batenburg, L. M. J. (2010). *J. Appl. Cryst.* **43**, 70–82.

[bb12] Sheldrick, G. (2009). *TWINABS*. University of Göttingen, Germany.

[bb13] Thompson, M. C. (2017). *Identifying and Overcoming Crystal Pathologies: Disorder and Twinning*, ch. 8, *Protein Crystallography: Methods and Protocols* in *Methods in Molecular Biology*, Vol. 1607. Clifton: Springer. 10.1007/978-1-4939-7000-1_8 28573574

[bb14] Welberry, T. R., Heerdegen, A. P., Goldstone, D. C. & Taylor, I. A. (2011). *Acta Cryst.* B**67**, 516–524. 10.1107/S0108768111037542 22101541

