# Supplementary material for: Crystal structure of the second extracellular domain of human tetraspanin CD9: twinning and diffuse scattering
Source: IUCrdata. 2022 Sep 23;7(Pt 9):x220852. doi: 10.1107/S2414314622008525 (PMC9635431; doi:10.1107/S2414314622008525)
Supplement: Supplementary file 2 [file x-07-x220852-img_check.pdf]

checkImgCIF report

Powered by https://github.com/jamesrhester/ImgCIFHandler.jl

Sample image

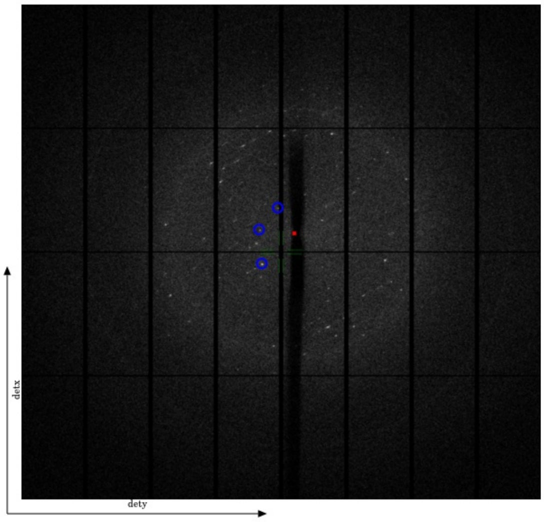

Laboratory frame

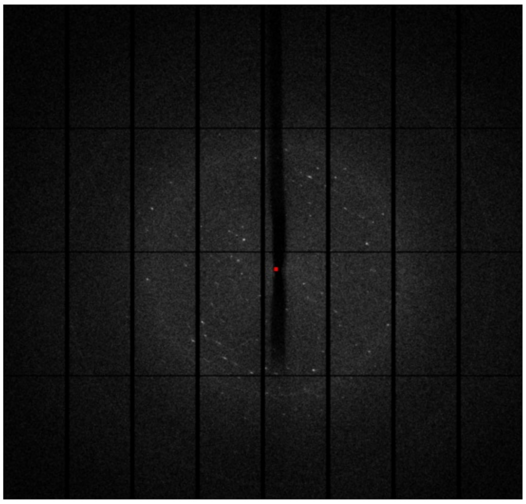

Original

```
 ImgCIF checker version 2022-08-04

Running checks (no image download)
========================================


Testing: Required items: PASS

Testing: Data source: PASS

Testing: Axes defined: PASS

Testing: Our limitations: PASS

Testing: Detector translation: PASS

Testing: Scan range: PASS

Testing: All frames present: PASS
   All frames present and correct for SCAN1

Testing: Detector surface axes used properly: PASS

Testing: Pixel size and origin described correctly: PASS

Testing: Check calculated beam centre: PASS

Testing: Check principal axis is aligned with X: PASS

Testing: All archives are accessible: PASS

Running checks with downloaded images
========================================


Testing image 4: Image type and dimensions: PASS
```

Testing image 4: Overloaded values present: PASS

====End of Checks====
